# The Efficacy of Neoadjuvant Chemotherapy for HER-2-Positive Locally Advanced Breast Cancer and Survival Analysis

**DOI:** 10.1155/2017/1350618

**Published:** 2017-09-19

**Authors:** Wei Zhang, Huan Tian, Shi-hong Yang

**Affiliations:** Department of Breast Surgery, The Second Affiliated Hospital of Guangzhou University of Chinese Medicine and Guangdong Provincial Hospital of Chinese Medicine, Da De Lu No. 111, Guangzhou, Guangdong 510120, China

## Abstract

To provide reference data, we retrospectively investigated the effects of neoadjuvant chemotherapy (NAC) on 119 patients with HER-2^+^ locally advanced breast cancer, treated from November 2010 to July 2016, with respect to influencing factors and survival. They were divided into the pathological complete response (pCR; *n* = 15; 12.6%) and non-pCR (*n* = 104; 87.4%) groups. We used *Χ*^2^ and logistic tests to the analyze effect and influencing factors. Survival rate was analyzed by the Kaplan-Meier method and Log-rank test. We lost 12 patients (including 1 pCR patient) and followed 107 patients, of whom 31 (all in the non-pCR group) had local recurrences or distant metastasis. The two groups significantly differed in 3-year disease-free survival (pCR group: 100%; non-pCR group: 59.0%; *P* = 0.039); pCR was significantly affected by histological grade, PR status, Ki67 index, primary tumor size, clinical staging, and number of trastuzumab cycles. The model was tested, and the difference was statistically significant (*Χ*^2^ = 31.938, *P* = 0.032). Patients with HER-2^+^ locally advanced breast cancer with pCR responses to NAC have improved prognoses. Patients without pCR have increased risk for relapse. The use of a combination of NAC, such as trastuzumab and chemotherapy, and more cycles should be considered to increase the likelihood of pCR.

## 1. Introduction

Breast cancer is the most common cancer in Chinese women [1]; of these cancers, 20–25% have overexpressed or gene amplified human epidermal growth factor receptor 2 (HER-2) [2], a subtype with high malignancy, distant metastasis rate and mortality, and relatively short disease-free survival (DFS) and overall survival (OS) [3, 4] and, therefore, a high need for clinical attention. For locally advanced breast cancer, neoadjuvant chemotherapy (NAC) is a standard treatment. Patients who attain pathological complete responses (pCR) to NAC have significantly longer OS and DFS [5]. If response (pCR or otherwise) can be predicted before undergoing NAC, NAC efficacy and patients' overall management can be improved [6]. This study retrospectively analyzed the efficacy and influencing factors of NAC on patients with HER-2^+^ breast cancer treated in our hospital.

## 2. Materials and Methods

### 2.1. Patients

We retrospectively investigated the clinical status of 119 women with HER-2^+^ locally advanced breast cancer who underwent treatment from November 2010 to July 2016, at the Guangdong Provincial Hospital of Chinese Medicine. We included those (a) with invasive cancer pathologically diagnosed by bud needle biopsy before chemotherapy; (b) with HER-2^+^ subtype (per 2013 St. Gallen standard with immunohistochemical (IHC) testing); (c) with clinical TMN stage II-III disease (per American Joint Committee on Cancer, 7th edition); and (d) who underwent at least two NAC courses. We excluded patients with (a) incomplete clinical data; (b) distant metastasis; (c) bilateral breast cancer; (d) antitumor treatment before NAC; or (e) a history of organ transplantation or other major organ diseases before NAC.

### 2.2. Immunohistochemical Methods and Results to Determine

Breast biopsy specimens were examined by automated immunohistochemistry. Specimens were embedded in paraffin and cut into 4 *μ*m sections.

For estrogen receptor (ER) and progesterone receptor (PR), ≥1% of cells with strongly stained tumor nuclei were considered positive, and <1% was negative. For HER-2, IHC detection of HER-2+++ was considered positive, and 0-1+ was considered negative; ++ required verification through fluorescence in situ hybridization (FISH), with 2.2 gene copies (per FISH) considered positive for *HER-2* amplification. For Ki67, positive cells had IHC-stained light yellow to brown nuclei, examined at 400x light microscopy in 10 randomly selected fields; 500 cells were counted in each field, and the ratio of Ki67-positive cells to total cells was calculated.

### 2.3. Treatment

All patients were diagnosed with invasive cancer by biopsy before chemotherapy, including breast ultrasonography and breast mammography to ascertain breast mass size and the presence of axillary lymph node metastasis, chest X-ray, abdominal ultrasound, and whole body bone scan to exclude distant metastases. We selected from the following options according to the US National Cancer Comprehensive Network (NCCN) guidelines: (1) docetaxel plus epirubicin plus cyclophosphamide (TEC); (2) epirubicin and cyclophosphamide sequential docetaxel or paclitaxel (every 2 weeks) with or without trastuzumab (EC-T/P ± H); (3) docetaxel plus carboplatin plus trastuzumab (TCH); and (4) replacement scheme: docetaxel plus cyclophosphamide (TC), vinorelbine plus cisplatin (NP), and vinorelbine plus trastuzumab (NH). The above regimens were intravenous infused every 3 weeks, with an interval of 2–4 weeks from the end of chemotherapy to surgery. The presurgical regimen was continued after surgery if NAC was effective; postoperative chemotherapy cycles were based on preoperative chemotherapy cycles, for a total of 6–8 cycles. The remaining treatment programs were in accordance with NCCN guidelines.

### 2.4. Clinical Efficacy Evaluation Criteria

According to Response Evaluation Criteria in Solid Tumors (RECIST; 1.1 edition), complete remission (CR) refers to the disappearance of all known lesions, maintained for more than 4 weeks; partial remission (PR) refers to the sum of the largest single diameter of the tumor reduced by more than 30% and maintained for more than 4 weeks; progressive disease (PD) refers to the sum of the maximum single diameter of the tumor increased by more than 20% or a new lesion; and stable disease (SD) refers to disease that does not meet the criteria for CR, PR, or PD, maintained for more than 4 weeks. Efficacy was evaluated every 2 cycles.

### 2.5. Postoperative Pathological Evaluation Criteria

We used the Miller-Payne scoring system in which 1 was defined as pathologically ineffective (SD), 2–4 as partial pathological relief (PR), and 5 as pCR. We also used the residual cancer burden (RCB) evaluation system, in which RBC III was considered pathologically ineffective (SD), RBC I-II as partial pathological relief (PR), and RBC 0 as pCR. We defined pCR as no residual invasive cancer cells in the primary tumor and regional lymph nodes, but some could be left over in in situ cancer. Our pathologically effective rate included both pCR and PR.

### 2.6. Follow-Up

From the first day of the first chemotherapy, all patients were followed up in December 2016, with follow-up information obtained through outpatient interviews or telephone. Recurrence refers to clinical and histological findings of ipsilateral breast or regional lymph node recurrence. Distant metastasis refers to clinical and imaging studies showing distant metastases. DFS was counted from the first day of the first chemotherapy to the date of first recurrence or metastasis or the last follow-up time.

### 2.7. Statistical Analysis

We used the SPSS 17.0 statistical software package to establish the database and statistical analysis. We calculated the percentages and rates of the enumeration data. Results were compared using the *Χ*^2^ test or Fisher's exact test. Multivariate analysis used unconditional stepwise logistic regression analysis. Survival was analyzed by the Kaplan-Meier method, using log-rank tests to draw survival curves; *P* < 0.05 was considered significant.

## 3. Results

### 3.1. Clinicopathological Characteristics and NAC in HER-2^+^ Breast Cancer

Of the 119 patients with HER-2^+^ breast cancer, the median onset age was 53 years (range: 28–81 [51.14 ± 9.92] years). Their clinicopathological characteristics are shown in Table 1. They were divided into the pCR group (*n* = 15; 12.6%) and the non-pCR group (*n* = 104; 87.4%). All 119 cases could be evaluated, and the operation was performed after NAC. But 1 patient of HR^+^HER-2^+^ did not undergo an operation because CT showed lung metastasis during NAC; another 1 patient of HR^−^HER-2^+^ showed local progress during NAC and was treated with mastectomy and immediate breast reconstruction according to the patient's wishes.

With pCR as the grouping variable, the univariate analysis found that PR status, Ki67 index, presurgical primary tumor size, clinical stage, and number of trastuzumab cycles were correlated with pCR (*P* = 0.032, 0.040, 0.001, 0.035, and 0.044, resp.; Table 1). Nonconditional logistic regression analysis showed that the model was significant (*Χ*^2^ = 31.938, *P* = 0.032). Logistic multivariate regression analysis showed that pCR was positively correlated with histological grade and Ki67 index (*P* = 0.023 and 0.048, resp.; OR: 7.250 and 95% CI: 1.313–40.032 for histological grade; OR: 4.971 and 95% CI: 1.014–24.376 for Ki67 index; Table 2).

### 3.2. HER-2^+^ Breast Cancer Subgroups and Prognosis

**Figure 1:**
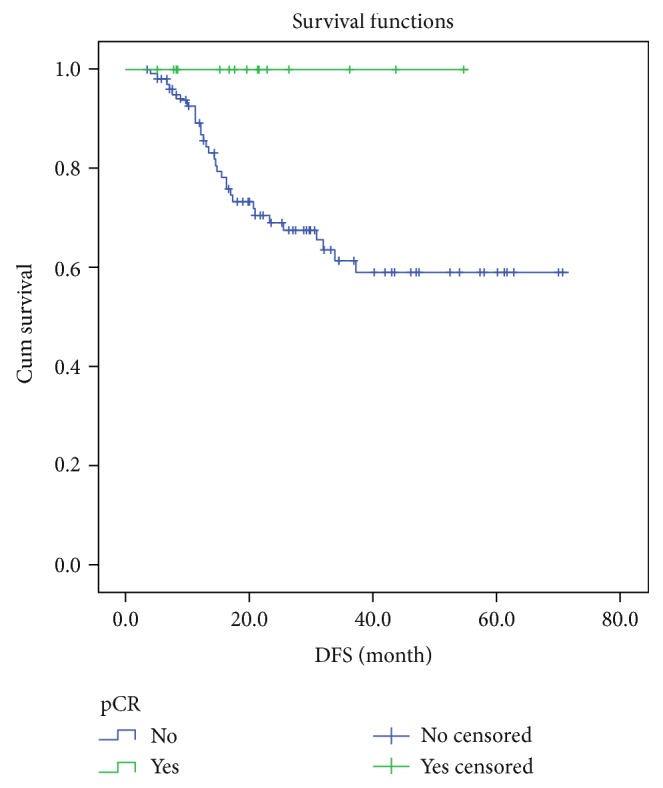
Kaplan-Meier 3-year DFS estimation in pCR and non-pCR patients.

**Figure 2:**
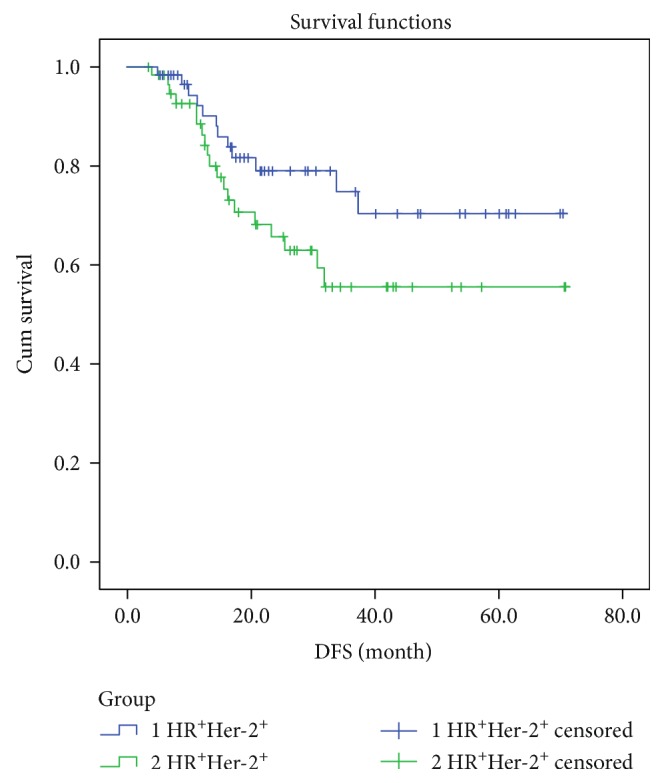
Kaplan-Meier 3-year DFS estimation in HR^+^ and HR^−^ patients.

We lost 12 patients to follow-up (including 1 patient in the pCR group) and followed 107 patients for a median of 20.6 months (range: 3.4–70.8 [28.86 ± 18.71]). A total of 31 patients, all in the non-pCR group, had local recurrence or distant metastasis. The two groups significantly differed in 3-year DFS (pCR group: 100%; non-pCR group: 59.0%; *P* = 0.039; Figure 1). Among the 31 patients with local recurrence or distant metastasis, 12 patients had tumors that expressed ER and/or PR (HR^+^), and 19 were HR^−^; their 3-year DFS did not significantly differ (HR^+^ group: 70.4%; HR^−^ group: 55.6%; *P* = 0.110; Figure 2).

## 4. Discussion

Neoadjuvant chemotherapy is part of the overall treatment of breast cancer and can increase long-term survival among patients who show pCR; pCR has therefore become a critical indicator for both the curative effect of NAC and also prognosis [7]. In this study, almost all of the pCR patients survived without disease, whereas only 59.0% of those who did not achieve pCR enjoyed DFS at 3 years.

HER-2^+^ breast cancer is highly malignant and has a poor prognosis; however, the latter has been significantly improved with the availability of trastuzumab, which has been widely reported to improve the pCR rate from 30.3% to 65% when used as part of a NAC regimen [8–11]. Therefore, NCCN, American Society of Clinical Oncology, European Society for Medical Oncology, and other major guidelines and expert consensus groups suggest that HER-2^+^ breast cancer treatments include trastuzumab-based NAC regimens. The pCR rate in this setting varies in different studies, which may be related to the number of cycles of NAC therapy, with higher pCR rates associated with more cycles. However, the present study indicates that, while 4–6 cycles give a relatively high pCR rate, more than 8 cycles of trastuzumab adversely affect pCR. Therefore, the author do not recommend giving more cycles of NAC in pursuit of a higher pCR rate. However, NAC cycles may be appropriately extended in the premise of effective treatment. The total pCR rate of this study was 12.6%, which significantly differed from those of international clinical studies, because 51.3% (61/119) of our patients did not use trastuzumab-based NAC. Therefore, to increase the pCR rate, trastuzumab-based NAC should be considered.

The current view is that HR^−^ tumors are more chemosensitive than HR^+^ tumors but have worse prognoses. In this study, rates of pCR by HR status were HR^+^ (*n* = 61), 18% (11/61), and HR^−^ (*n* = 58), 6.9% (4/58). Three-year DFS rates did not significantly differ (HR^+^: 70.4%; HR^−^: 55.6%; *P* = 0.110). The DFS of HR^+^ patients was slightly better than that of the HR^−^ group, because the use of adjuvant endocrine therapy in HR^+^ patients reduces recurrence risk. The differences between our results and those of other studies (1) may be related to our small sample size and short follow-up time (2) but might also reflect differences in pCR rates by ER and PR status considered separately. Our univariate analysis showed that the pCR rates were ER^+^ 15.3% and ER^−^ 10.0% (*P* = 0.388) and PR^+^ 21.4% and PR^−^ 7.8% (*P* = 0.032), which indicates that the pCR rate of HR^+^ patients was higher and the PR status had some impact on pCR. This is consistent with Peintinger et al. [12] who found that HR^+^ breast cancer patients had higher pCR rates but contrast with others' results [13, 14]. The predictive value of HR status in HER-2^+^ breast cancer NAC is not clear. (3) Larger tumors, those with lymph node involvement and higher-stage disease, are less likely to achieve pCR and are associated with shorter DFS [15, 16]. In this study, initial tumor sizes were significantly larger among HR^−^ patients (for cT1, cT2, cT3, and cT4: 0 [0%], 11 [19%], 23 [39.6%], and 24 [41.4%], resp.) than among HR^+^ patients (for cT1, cT2, cT3, and cT4: 1 [1.6%], 25 [41%], 23 [37.7%], and 12 [19.7%], resp.; *P* = 0.016). Before treatment, HR^−^ patients had larger primary tumors, later-stage disease, and heavier tumor burden than HR^+^ patients, so their treatment was more difficult and their remission rate was lower. Possibly because of the lower pCR rate, the DFS rate in the HR^−^ group was not significant. The importance of early detection, early diagnosis, and early treatment is also indicated.

This study was limited by its small sample size, short follow-up time, and single-institution design. Our results should be verified by a larger-scale study through several institutions and over a longer follow-up time.

In summary, HER-2^+^ breast cancer patients with a pCR after NAC have an improved prognosis, but those without a pCR have increased risk for relapse. High histological grade, high Ki67 expression, PR^+^ status, smaller primary tumor, lower clinical stage, and number of trastuzumab-based cycles were correlated with pCR and had some predictive value. To increase the likelihood of pCR, increasing the number of trastuzumab-based NAC cycles (such as a combination of trastuzumab and chemotherapy) should be considered.

## Figures and Tables

**Table 1:** Univariate analysis of factors associated with pCR.

Characteristic		Non-pCR	pCR	*Χ* ^2^	*P*
Age (year)	≤40	15	2	0.013	0.910
	>40	89	13		

Histological type	Invasive ductal	99	15	0.753	0.386
	Invasive papillary	5	0		

Histological grade	2	79	9	1.734	0.188
	3	25	6		

ER status	Negative	54	6	0.745	0.388
	Positive	50	9		

PR status	Negative	71	6	4.587	0.032
	Positive	33	9		

Ki67	≤30%	70	6	4.2	0.040
	>30%	34	9		

Primary tumor size preoperation	cT1	0	1	15.748	0.001
	cT2	27	9		
	cT3	42	4		
	cT4	35	1		

Regional lymph node preoperation	cN0	3	0	1.38	0.710
	cN1	23	5		
	cN2	28	3		
	cN3	50	7		

Clinical stage preoperation	IIA	2	0	10.368	0.035
	IIB	5	4		
	IIIA	35	4		
	IIIB	11	0		
	IIIC	51	7		

Treatment regimen	TEC	49	5	2.784	0.733
	EC-T	4	1		
	TCH	13	2		
	EC-TH	26	6		
	Replacement single chemotherapy	6	0		
	Replacement chemotherapy + H	6	1		

Trastuzumab cycle preoperation	0	59	6	11.418	0.044
	2-3	13	1		
	4	19	6		
	5	0	1		
	6	11	1		
	8	2	0		

**Table 2:** Multivariate analysis of factors associated with pCR.

Characteristic	B	SE	Wald	Df	*P*	Exp (B)	95% CI for Exp (B)	
							Lower	Upper
Histological grade	1.981	0.981	5.165	1	0.023	7.250	1.313	40.032
Ki67	1.604	0.811	3.908	1	0.048	4.971	1.014	24.376
Constant	−18.326	13458.80	0.000	1	0.999	0.000	—	—
